# Prescribing Exercise and Lifestyle Training for High Risk Women in Pregnancy and Early Post-partum—Is It Worth It?

**DOI:** 10.1371/journal.pmed.1002093

**Published:** 2016-07-26

**Authors:** Jane E. Norman, Rebecca M. Reynolds

**Affiliations:** 1 Tommy’s Centre for Maternal and Fetal Health, MRC Centre for Reproductive Health, University of Edinburgh, Edinburgh, United Kingdom; 2 British Heart Foundation Centre for Cardiovascular Research, University of Edinburgh, Edinburgh, United Kingdom

## Abstract

In a Perspective, Jane Norman and Rebecca Reynolds discuss two randomized controlled trials aimed at testing behavioral interventions for women at risk of gestational diabetes.

The positive effects of exercise on health are supported by systematic reviews of longitudinal studies and are widely accepted by clinicians [1]. In pregnancy, current advice from the United Kingdom’s National Health Service (NHS) Choices and National Institute for Health and Care Excellence, as well as the American College of Obstetricians and Gynecologists (ACOG), is that exercise is more likely to be beneficial than harmful, with ACOG advising that “an exercise programme [with] a goal of moderate intensity exercise for at least 20–30 minutes per day should be developed with the patient” [2].

The research paper by Trine Moholdt and colleagues in *PLOS Medicine* describes a randomized trial on the effects of supervised exercise training in obese pregnant women [3]. Women in the exercise group were offered exercise sessions supervised by a physical therapist at a hospital three times weekly from baseline (12–18 weeks) until delivery. The sessions lasted 60 minutes and included 35 minutes of walking/jogging with additional resistance training. Women were also advised to follow 50 minutes of a home exercise program at least once weekly. The control group was offered routine care, which included “information about healthy eating and a healthy lifestyle.”

Exercise training had no effect on the proportion of women reporting being physically active each day in late pregnancy, with rates being just over 60% in both groups. Additionally, the estimated energy expenditure during each session was low (around 400 kcal/session), and only 50% of participants attended the equivalent of 50% of the sessions or more. However, more women in the exercise intervention group reported exercise training in late pregnancy (77% versus 23%).

There was no effect of the intervention on the preplanned primary outcome of mean weight gain in the two groups: 10.5 kg (95% CI 8.9–12.0) for women in the exercise group and 9.2 kg (6.8–11.6) for those in the control group. Nor did the intervention reduce the proportion of women with weight gain above Institute of Medicine recommendations (5–9 kg for women with a body mass index [BMI] ≥ 30 kg/m^2^). Amongst the secondary outcomes, a lower rate of gestational diabetes mellitus (GDM) was reported in the exercise group: this apparent difference reached statistical significance for GDM diagnosed by WHO criteria (6.1% versus 27.3%) but not by the International Association of Diabetes in Pregnancy Study Groups’ criteria. These findings chime with a reduced risk of GDM reported for lifestyle interventions in high-risk women with obesity or a previous history of GDM [4].

The limitations of Moholdt and colleagues’ trial are the lower than planned sample size (91 rather than 150 women) and the inclusion of a small number of women (<10% overall) who were overweight rather than obese. Nevertheless, these data complement those from a Cochrane systematic review of diet or exercise interventions (or both) in pregnancy, which demonstrated a small but significant reduction in the risk of excessive weight gain (relative risk 0.80 [95% CI 0.73–0.87]) but found no effect on mean weight gain in the majority of studies [5].

What are the clinical implications of Moholdt and colleagues’ study? It is tempting to be nihilistic and to point out that not only did the intervention fail to achieve its stated aim of reducing mean weight gain but that it is likely to have been prohibitively expensive: provision of three supervised exercise sessions per week for up to 30 weeks is a major financial undertaking. A cost-effectiveness analysis was not performed as part of this study but is unlikely to show benefit: during the time horizon of the study, even the reduction in GDM is unlikely to offset the costs of the intervention.

So should we stop recommending exercise and/or stop testing exercise interventions in obese pregnant women? The answer might be not that the intervention of exercise (supervised or otherwise) is ineffective but that inappropriate outcomes are being used to assess utility in pregnancy.

For the baby, maternal exercise in pregnancy, with or without a reduction in maternal GDM, might minimize the adverse programming effects of maternal obesity on long-term health of the offspring. We have shown that maternal obesity was associated with a 35% increased risk of premature all-cause mortality on offspring by the age of 50 years—even a modest reduction in this risk would have major health and economic benefits for society [6].

For the mother, exercise may not reduce mean weight gain in the index pregnancy, but if the lifestyle change is maintained, it might mean that she enters the next pregnancy with a lower BMI. Additionally, an exercise intervention may have benefits beyond the woman’s childbearing years: a modest fall in bodyweight will reduce the lifelong risk of cardiovascular disease [7,8].

In a separate research study also published in *PLOS Medicine* (the Mothers after Gestational Diabetes in Australia [MAGDA] trial), Sharleen O'Reilly and colleagues focused on this lifelong risk to the mother and evaluated a postnatal lifestyle intervention program in women diagnosed with GDM during pregnancy [9]. Five hundred and seventy-three women were recruited, with half randomized to the intervention and the other half to usual care. The intervention (diet and exercise advice) was delivered in a group exercise (five in total), together with one individual session and two phone sessions, all over a period of 3 months. The majority of the participants (66%) were overweight or obese.

As in the study by Moholdt and colleagues, engagement by participants was suboptimal in O’Reilly and colleagues’ trial, with only 53% attending one or more sessions. According to the trial’s coprimary endpoints, the intervention had no significant effect on fasting blood glucose, although a small but significant difference in weight gain was observed over a 12-month period (between-group difference of −0.95 kg, 95% CI −1.87 to −0.04), with the lower weight gain recorded in the intervention group. As the authors mention, such an intense lifestyle intervention may not be cost-effective compared with annual screening for diabetes with intervention in those with impaired glucose tolerance. Additionally, both of these studies highlight the need to engage patients in study design in order to optimize adherence to lifestyle interventions.

We note that Moholdt and colleagues plan to follow-up the mothers and babies recruited to their study, to determine longer-term effects [10]. We would urge them and other investigators conducting similar studies, including O’Reilly and colleagues, to do so. Given the major lifelong adverse effects of maternal obesity for both the mother and the baby, effective therapeutic interventions are desperately needed [11]. Guidelines promoting exercise in pregnancy are likely to do more good than harm, although they are not strongly supported by current evidence. Metformin has been shown to be effective in reducing the risk of future diabetes in women with a history of GDM, but lifestyle interventions are more effective in obese women without a history of GDM [12]. Hence, lifestyle interventions offer the best promise for prevention and should not be abandoned because of limited effectiveness or lack of effectiveness as judged by short-term surrogate outcomes.

## Abbreviations

ACOG: American College of Obstetricians and Gynecologists
BMI: body mass index
GDM: gestational diabetes mellitus
MAGDA: Mothers after Gestational Diabetes in Australia
NHS: National Health Service

## References

[1] Reiner M, Niermann C, Jekauc D, Woll A. Long-term health benefits of physical activity—a systematic review of longitudinal studies. BMC Public Health. 2013;13:813 10.1186/1471-2458-13-813 24010994 PMC3847225

[2] ACOG Committee Opinion No. 650: Physical Activity and Exercise During Pregnancy and the Postpartum Period. Obstet Gynecol. 2015;126(6):e135–142. 10.1097/AOG.0000000000001214 . 26595585

[3] Gamaes K, Morkved S, Salvesen O, Moholdt T. Exercise Training and Weight Gain in Obese Pregnant Women: A Randomised Controlled Trial (ETIP Trial). PLoS Med. 2016;13: e1002079 10.1371/journal.pmed.1002079 27459375 PMC4961392

[4] Koivusalo SB, Rono K, Klemetti MM, Roine RP, Lindstrom J, Erkkola M, et al Gestational Diabetes Mellitus Can Be Prevented by Lifestyle Intervention: The Finnish Gestational Diabetes Prevention Study (RADIEL): A Randomized Controlled Trial. Diabetes Care. 2016;39(1):24–30. 10.2337/dc15-0511 . 26223239

[5] Muktabhant B, Lawrie TA, Lumbiganon P, Laopaiboon M. Diet or exercise, or both, for preventing excessive weight gain in pregnancy. Cochrane Database Syst Rev (Online). 2015;(6):CD007145 10.1002/14651858.CD007145.pub3 . 26068707 PMC9428894

[6] Reynolds RM, Allan KM, Raja EA, Bhattacharya S, McNeill G, Hannaford PC, et al Maternal obesity during pregnancy and premature mortality from cardiovascular event in adult offspring: follow-up of 1 323 275 person years. BMJ. 2013;347:f4539 10.1136/bmj.f4539 23943697 PMC3805484

[7] Gooding HC, Shay CM, Ning H, Gillman MW, Chiuve SE, Reis JP, et al Optimal Lifestyle Components in Young Adulthood Are Associated With Maintaining the Ideal Cardiovascular Health Profile Into Middle Age. J Am Heart Assoc. 2015;4(11): pii: e002048. 10.1161/JAHA.115.002048 26514160 PMC4845225

[8] Berrington de Gonzalez A, Hartge P, Cerhan JR, Flint AJ, Hannan L, MacInnis RJ, et al Body-mass index and mortality among 1.46 million white adults. N Engl J Med. 2010;363(23):2211–2219. 10.1056/NEJMoa1000367 21121834 PMC3066051

[9] O'Reilly S, Dunbar J, Versace V, Janus E, Best JD, Carter R, et al Mothers After Gestational Diabetes in Australia (MAGDA): A Randomized, Controlled Trial of a Postnatal Prevention Program. PLoS Med. 2016;13: e1002092 10.1371/journal.pmed.1002092 27459502 PMC4961439

[10] Moholdt TT, Salvesen K, Ingul CB, Vik T, Oken E, Morkved S. Exercise Training in Pregnancy for obese women (ETIP): study protocol for a randomised controlled trial. Trials. 2011;12:154 10.1186/1745-6215-12-154 21682869 PMC3148988

[11] Lee KK, Raja EA, Lee AJ, Bhattacharya S, Bhattacharya S, Norman JE, et al Maternal Obesity During Pregnancy Associates With Premature Mortality and Major Cardiovascular Events in Later Life. Hypertension. 2015;66(5):938–944. 10.1161/HYPERTENSIONAHA.115.05920 . 26370890

[12] Aroda VR, Christophi CA, Edelstein SL, Zhang P, Herman WH, Barrett-Connor E, et al The Effect of Lifestyle Intervention and Metformin on Preventing or Delaying Diabetes Among Women With and Without Gestational Diabetes: The Diabetes Prevention Program Outcomes Study 10-Year Follow-Up. J Clin Endocrinol Metab. 2015:jc20143761. 10.1210/jc.2014-3761 . 25706240 PMC4399293

